# Long-Term Effects of the Combined Application of Organic and Inorganic Fertilizers on Soil Fertility, Structural Stability, and Rice Productivity in Cool Rice-Growing Regions of Northeast China

**DOI:** 10.3390/plants15070993

**Published:** 2026-03-24

**Authors:** Yuwei Xin, Benqi Yue, Xin Zhao, Shanlong Li, Tao Li, Jian Ren, Yutong Li, Yutong Yang, Wenze Li, Kokyo Oh, Tiehua Cao, Xuanhe Liang

**Affiliations:** 1 Institute of Agricultural Resources and Environment, Jilin Academy of Agricultural Sciences (Northeast Agricultural Research Center of China), Changchun 130033, China; xywyykl2021@163.com (Y.X.); zhaoxin8401@163.com (X.Z.); lishanlong05@163.com (S.L.); ndlitao@126.com (T.L.); renj849@nenu.edu.cn (J.R.); lyutong233@126.com (Y.L.); yyt15846896885@139.com (Y.Y.); 15143552399@163.com (W.L.); 2 Faculty of Agronomy, Jilin Agricultural University, Changchun 130118, China; 3 Jilin Province Green Food Office, Changchun 130022, China; 13756926009@139.com; 4 Agriculture College, Yanbian University, Yanji 133002, China; 5 Center for Environmental Science in Saitama, Saitama 347-0115, Japan; o.kokyo@pref.saitama.lg.jp

**Keywords:** combined application of organic and inorganic fertilizers, soil aggregates, soil fertility, nitrogen use efficiency, rice productivity, paddy fields ecological environment

## Abstract

To investigate the long-term effects of combined organic and inorganic fertilizer application on the structural stability and fertility of soil in paddy fields located in the cool northeastern region of China, a long-term fixed-site experiment was initiated in 2017. The experiments included the following five treatments: 100% conventional chemical fertilizer NPK (CK), conventional PK fertilizer without N fertilizer (T1), 30% organic N and 70% chemical N fertilizers with conventional PK fertilizer (T2), 50% organic N and 50% chemical N fertilizers with conventional PK fertilizer (T3), and 100% organic N fertilizer (T4). Notably, the total amount of fertilizer applied remained consistent across treatment groups. The results revealed that the combination of organic and inorganic fertilizers significantly increased rice yields and nitrogen use efficiency, with the T3 treatment performing the best. Compared with CK, T3 resulted in a 24.26% greater rice yield, and it increased the nitrogen agronomic efficiency by 71.05%. There were no significant differences among the treatment groups in terms of the proportions of soil aggregates larger than 2 mm or smaller than 0.053 mm. Nitrogen fertilizer application reduced the proportion of 0.053–0.25 mm aggregates and promoted the formation of predominantly 0.25–2 mm aggregates. However, the different organic–inorganic combinations did not cause significant differences in soil aggregate structure or stability. Compared with the CK treatment, the application of both organic and inorganic fertilizers increased soil organic matter content, decreased N_2_O emissions, and increased soil catalase activity. In summary, the application of 50% organic N and 50% chemical N fertilizers with conventional PK fertilizer (T3) was determined to be the optimal combination for achieving high and stable rice yields in the cool northeastern region of China while increasing the structural stability and fertility of the soil.

## 1. Introduction

Rice (*Oryza sativa* L.), which is China’s largest cereal crop, plays a crucial role in ensuring national food security, with more than 60% of the population relying on rice as a staple food [1]. Jilin Province, which is a major area for high-quality japonica rice production and the core zone of the commodity grain base in Northeast China, maintains a stable annual rice planting area of more than 666,700 hectares. However, long-term issues such as excessive chemical fertilizer use and insufficient organic fertilizer input have led to a decline in soil organic matter content, deterioration of physicochemical properties, and degradation of ecological functions in the paddy fields of this region. These problems not only limit the synergistic improvement in rice yields and quality but also exacerbate low fertilizer use efficiency and agricultural nonpoint source pollution, severely threatening the sustainable utilization of black soil resources in Northeast China.

The combined application of organic and inorganic fertilizers, which is a model of fertilization that balances high yields and soil fertility, not only completely relies on the fast-acting effect of chemical fertilizers to meet nutritional needs during key rice growth periods but also supplements soil organic matter content and improves soil structural stability through the use of organic fertilizers, resulting in long-term soil enrichment [2,3]. The application of an appropriate proportion of organic fertilizer as a substitute for chemical fertilizer can effectively balance the rapid nutrient release of chemical nitrogen fertilizers with the slow, stable release of nutrients from organic fertilizers, meeting the nutritional needs of crops at different stages of growth [4,5]. The application of excessive amounts of organic fertilizer may lead to an oversupply of nitrogen in the later growth stages in paddy fields, causing excessive vegetative growth, delayed maturation, and reduced grain setting, ultimately leading to decreased yields [6]. Numerous studies have shown that when the nitrogen content from organic fertilizer accounts for 20–50% of the total nitrogen content, rice yields significantly increase, but if the organic nitrogen content exceeds 60% of the total content, yields may decrease [7,8,9,10,11]. Combining fertilizers in an appropriate ratio (typically with organic nitrogen substitution rates between 25% and 50%) can also significantly reduce soil bulk density, increase soil porosity, and promote the formation of water-stable aggregates [12,13,14,15,16]. A substitution ratio that is too high may result in insufficient decomposition of organic matter, which is unfavorable for the structural stability of the soil; however, a substitution ratio that is too low leads to negligible improvement, and the physical structure of the soil cannot be sustainably optimized. Research has shown that a 30–60% substitution of organic nitrogen can maintain high crop yields while effectively increasing soil organic carbon and total nitrogen contents [17,18]. The appropriate input of organic matter provides effective carbon sources and nutrients for soil microorganisms, significantly increasing microbial biomass and the activity of key soil enzymes such as urease, sucrase, and catalase [19]. However, a proportion of organic fertilizer that is too high may lead to a carbon–nitrogen imbalance, suppressing the activity of certain microorganisms.

The combination of organic and inorganic fertilizers not only improves arable land quality but also has significant effects on regulating greenhouse gas emissions and heavy metal environmental risks. Achieving an appropriate ratio of organic to inorganic fertilizers can enhance the soil structure, reduce the soil respiration intensity, and increase the organic carbon sequestration capacity, thereby supporting carbon sequestration and reducing emissions [20]. Conversely, improper fertilizer application, especially excessive organic fertilizer input, can promote methane (CH_4_) and nitrous oxide (N_2_O) emissions under anaerobic conditions, exacerbating the greenhouse effect [21]. Studies have shown that when the substitution rate of organic nitrogen is too high (exceeding 70%) or when the carbon-to-nitrogen ratio is imbalanced, denitrification is significantly enhanced, leading to increased N_2_O emissions [22,23]. In terms of the environmental risks associated with heavy metals, organic fertilizers can immobilize heavy metals through complexation and adsorption [24]. However, excessive contents of soluble organic matter may instead promote the mobilization and migration of heavy metals [25]. On the basis of previous research, an organic nitrogen substitution rate of 25–60% is generally considered to be an optimal range for balancing soil fertility improvement, heavy metal immobilization, and greenhouse gas mitigation.

To date, research on the application of both organic and inorganic fertilizers has mostly focused on warm rice-growing areas or short-term experiments. In cold rice-growing regions, such as northeastern black soil rice areas, where the average annual temperature is low, the growing season is short, and soil microbial activity is inhibited by low temperatures, long-term, location-specific studies remain markedly insufficient. Implementing the strategy of ‘increasing organic fertilizer and decreasing chemical fertilizer’ in cold rice-growing regions involves not only nutrient substitution but also a synergistic balance among nutrient supply rates, soil microecology, and crop uptake. In this study, on the basis of a long-term fertilization experimental platform in Longjing city, Jilin Province, Northeast China, the goal was to assess a cold-region japonica rice production system in Northeast China. We propose the following scientific hypothesis: under low-temperature conditions, an appropriate ratio of organic to inorganic fertilizers can achieve comprehensive benefits in terms of rice yield enhancement, soil conservation, and environmental risk mitigation over the long term. These findings are expected to provide theoretical support and technical guidance for scientific fertilization, green production, and black soil protection and utilization in rice production in Northeast China.

## 2. Materials and Methods

### 2.1. Overview of the Test Site

The trials were initiated in 2017. The test site is located within a field featuring a long-term rice trial at the Yanbian Academy of Agricultural Sciences in Longjing city, Jilin Province, China (42°46′ N, 129°26′ E). This area has a temperate continental monsoon climate, with an average annual temperature of 5.6 °C, an average annual precipitation totaling 549.3 mm, an annual accumulated temperature of 2800 °C, and an average frost-free period of 143 days. The soil type at the trial site is black soil. Before the trial, the basic soil fertility indicators included a total nitrogen concentration of 1.26 g kg^−1^, a total phosphorus concentration of 0.64 g kg^−1^, a total potassium concentration of 10.2 g kg^−1^, an alkaline hydrolyzed nitrogen concentration of 129.65 mg kg^−1^, an available phosphorus concentration of 14.8 mg kg^−1^, an available potassium concentration of 105.7 mg kg^−1^, an organic matter content of 2.89%, and a pH of 6.86.

**Figure 1:**
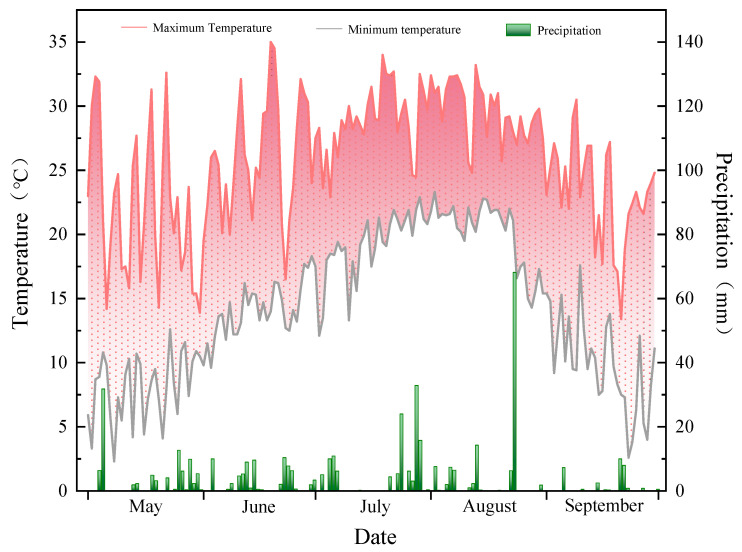
Temperature and precipitation conditions in Longjing city during the 2024 rice growth season. Note: The line chart in the figure shows temperature changes, while the bars show rainfall amounts.

Since the establishment of the experimental platform, each treatment has been continuously applied to the same experimental area over the long term, with the goal of creating stable and significantly different physicochemical and biological soil conditions and providing a mature and stable soil management context for subsequent rice cultivation. The data used in this study originated from the 2024 rice-growing season (transplanted on 28 May and harvested on 27 September), which was characterized by a cumulative temperature of 2533.1 °C and precipitation totaling 434.7 mm (Figure 1).

### 2.2. Test Materials

The rice variety used in this trial was *Jihong No. 6* (medium-maturing japonica rice), which was developed using *Jiyu Japonica* as the maternal parent and *Jiuyin No. 1* as the paternal parent. *Jihong No. 6* is suitable for cultivation in the rice-producing areas of Yanbian, Liaoyuan, and Songyuan in Jilin Province. The rice has the following features: dehulling rate ≥ 80%, whole milled rice rate ≥ 70%, and taste quality score of 86; thus, this variety meets the national Grade 2 standard for high-quality rice.

The test fertilizers included urea (46% N; Yunnan Yuntian Chemical Co., Ltd., Kunming, China); diammonium phosphate (containing 18% N and 46% P_2_O_5_; Yingkou Gangxing Fertilizer Co., Ltd., Yingkou, China); triple superphosphate (containing 46% P_2_O_5_; Hubei Fengle Ecological Fertilizer Co., Ltd., Zhongxiang, China); potassium chloride (containing 50% K_2_O; Qingshang Chemical (Jilin) Co., Ltd., Jilin, China); and commercial organic fertilizer (containing 40% organic matter, with activated nitrogen, phosphorus, and potassium contents of ≥5%, 3%, and 2%, respectively; Qingdao Haida Bio-Group Co., Ltd., Qingdao, China).

### 2.3. Experimental Design

The experimental setup was as follows. Five treatments that combined organic and inorganic fertilizers in different ratios were assessed: CK: 100% conventional chemical fertilizer NPK; T1: no N fertilizer, conventional PK fertilizer; T2: 30% organic N and 70% chemical N fertilizers, conventional PK fertilizer; T3: 50% organic N and 50% chemical N fertilizers, conventional PK fertilizer; and T4: 100% organic N fertilizer.

The fertilizer application rates were 150 kg hm^−2^ of pure nitrogen, 70 kg hm^−2^ of pure phosphorus, and 60 kg hm^−2^ of pure potassium. For all the treatments (except T4: 100% organic fertilizer), phosphorus and potassium fertilizers were applied once at the basal dose, whereas nitrogen fertilizer was applied at a basal dose: tillering: panicle ratio of 4:3:3. For the 100% organic N treatment, all nitrogen was applied as organic basal fertilizer, and P and K were derived entirely from the organic fertilizer; no additional chemical fertilizer was applied.

The experiments were conducted using a large-plot design, with each plot covering 1000 m^2^. Before the experiment was started, basic physical and chemical properties of soil throughout the entire experimental field were measured to ensure that the initial soil fertility, texture, and other key conditions were uniform across the large-area plots, and independent water and fertilizer management measures were applied to each treatment. Rice seeds were pregerminated and raised in a greenhouse nursery before being transplanted to the field on 28 May at a planting density of 30 cm × 13.3 cm; then, the rice was harvested on 27 September.

Tillage practices included autumn plowing to a depth of 18–20 cm, followed by autumn rotary plowing to a depth of 10–15 cm. In the spring, shallow flooding was applied without puddling, and the field was leveled with a harrow and irrigated with deep water. All the other management practices were conducted in accordance with conventional field management guidelines.

### 2.4. Sample Collection and Analysis

**Figure 2:**
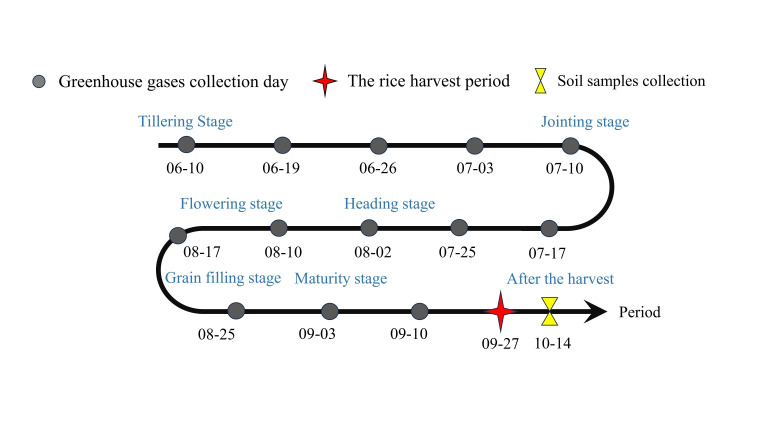
Sample collection and analysis timeline.

In this study, samples were collected and measured sequentially according to the timeline shown in Figure 2; the specific methods were as follows.

#### 2.4.1. Rice Yields and Nitrogen Fertilizer Indicators

After rice had matured, a representative 5 m^2^ area was selected in each plot (excluding edge effects and choosing the central area of the plot), and the rice was manually harvested, threshed, and naturally sun-dried to a moisture content of 14%. The actual yield was measured and converted to the yield per hectare, and the partial productivity of nitrogen fertilizer and nitrogen use efficiency values for each treatment were calculated [26].

#### 2.4.2. Soil Aggregates

After the rice was harvested, samples of in situ soil from the 0–20 cm plow layer were collected from each site treatment using the five-point sampling method. The collected soil samples were placed in rigid plastic boxes and returned to the laboratory. Visible plant and animal residues, stones, and other debris were removed, and the soil was gently broken along natural structural cracks into aggregates that were approximately 1 cm in size. The soil was then dried naturally in a cool, ventilated location for later use.

Soil aggregates were evaluated using the wet sieving method [27]. First, 100 g of air-dried soil was placed evenly on the top layer of an automatic shaking sieve (>2 mm, >0.25 mm, and >0.053 mm). The soil was moistened with distilled water at room temperature for 10 min before being subjected to vertical shaking with an amplitude of 5 cm at a frequency of 40 times per minute for 10 min. The aggregates on each sieve level were collected in aluminum boxes. Soil in the water bucket was first collected by sedimentation and centrifugation and then dried to a constant weight in a thermostatically controlled oven at 60 °C. The stability of the soil aggregates was evaluated by calculating the mean weight diameter (MWD), geometric mean diameter (GMD), and fractal dimension (D_v_).

#### 2.4.3. Soil Fertility Indicators

Soil samples were collected after the rice was harvested. Three samples were collected from each treatment site, and each sample was composed of 0–20 cm of topsoil collected using a five-point sampling method. After being brought back to the laboratory, the samples were immediately divided into two portions. One portion was air-dried, ground, and passed through 20- and 100-mesh sieves, and then, physicochemical properties of the soil (including pH, total nitrogen, total phosphorus, total potassium, alkaline hydrolyzable nitrogen, available phosphorus, available potassium, organic matter content, and heavy metal content, including Pb, Hg, and Cd content) were measured; the other portion was kept as a fresh sample at 4 °C, and it was used to measure urease and catalase activities. These two portions reflect the biological activity of the soil from the perspectives of nitrogen transformation and carbon metabolism and serve as key enzymatic indicators for evaluating the fertility of paddy fields. Urease is involved in nitrogen transformation, influencing crop nitrogen use efficiency and N_2_O emissions, whereas catalase reflects organic carbon decomposition and CO_2_/CH_4_ source–sink characteristics.

The soil pH was determined using the water–soil suspension potential method [28]. The total nitrogen content in the soil was measured by the Kjeldahl method. The total phosphorus content in the soil was determined using the acid-soluble molybdenum–antimony anticolorimetric method. The total potassium content in the soil was measured via flame photometry. The alkali-hydrolyzable nitrogen content in the soil was determined using the alkali diffusion method [29]. The available phosphorus content in the soil was measured by the NaHCO_3_ extraction technique followed by the molybdenum–antimony anticolorimetric method. The rapidly available potassium content in the soil was determined by NH_4_OAc extraction and flame photometry. The soil organic matter content was measured using the external heating method with potassium dichromate and concentrated sulfuric acid [30]. The concentrations of the heavy metals Pb and Cd in the soil were determined via atomic absorption spectrophotometry [31]. The Hg content in the soil was measured using atomic fluorescence spectrophotometry [32]. Catalase activity was determined through spectrophotometry [33]. Finally, urease activity was measured using the indophenol blue colorimetric method.

#### 2.4.4. Determination of Greenhouse Gases

Greenhouse gas (e.g., N_2_O, CH_4_, and CO_2_) emissions during the rice-growing season were measured via static chamber gas chromatography [34]. The dimensions of the static chamber were 60.5 cm × 35.5 cm × 40.0 cm; the outer layer was wrapped in aluminum foil to maintain a stable internal temperature, and a thermometer was installed inside the chamber to record the temperature in real time. The base measured 60.0 cm × 35.0 cm × 20.0 cm, and before sampling, a certain depth of clean water was added to the water tray to ensure chamber sealing. Gas samples (40 mL) were collected using a syringe and injected into gas collection bottles. The sampling period was from 10 June to 10 September, with a sampling frequency of once every seven days (delayed to the following day in the case of rainfall). The sampling covered the main phenological stages of rice, including the tillering stage, jointing stage, heading stage, flowering stage, grain-filling stage, and maturity stage, with a total of 13 sampling events completed. To obtain data that were representative of the day, all the sampling was conducted uniformly between 9:00 and 11:00 a.m. The specific method was as follows: The first sample was collected immediately after sealing the gas chamber, and additional samples were collected at 15 min intervals, for a total of four sampling points. Gas concentrations were determined using a gas chromatograph (Agilent 7890B, Agilent Technologies Co., Ltd., Santa Clara, CA, USA), and emission fluxes were calculated according to the formula. CO_2_ and CH_4_ concentrations were measured using an FID detector, and the N_2_O concentrations were measured using an ECD detector. All the processes were repeated three times.

#### 2.4.5. Formulas for Calculating Relevant Indicators

The PFPN and NUE after different fertilizer treatments were calculated as follows [35]:

(1) $$PEPN=\frac{Y}{F_{N}}$$

(2) $$NUE=\frac{Y_{F}-Y_{0}}{F_{N}}$$

where Y represents the crop yield, Y_F_ represents the crop yield in areas where nitrogen fertilizer was applied, Y_0_ represents the crop yield in areas without nitrogen fertilizer, and F_N_ represents the amount of nitrogen applied in units of kg hm^−2^.

The MWD [36], GMD [37], and D_v_ [38] of the soil aggregates were calculated using the following formulas to assess the stability of the soil aggregate structures after different treatments.

(3) $$MWD=\sum_{i=1}^{n}X_{i}W_{i}$$

(4) $$GMD=exp\left(\sum_{i=1}^{n}W_{i}lnX_{i}\right)$$

(5) $$\left(3-D_{V}\right)=lg\left[\frac{X_{i}}{X_{max}}\right]=lg\left[\frac{W_{\left(i\leq X_{i}\right)}}{W_{0}}\right]$$

where X_i_ represents the average diameter (mm) of the i-th particle size aggregate, W_i_ represents the percentage content (%) of the i-th particle size aggregate, X_max_ represents the average diameter (mm) of the largest particle size aggregate, W_(r<Xi)_ represents the weight (g) of aggregates smaller than X_i_, and W_0_ represents the total weight (g) of all soil particle sizes.

The greenhouse gas emission fluxes [34] from paddy fields were calculated using the following formula.

(6) $$F=\rho_{0}\times \frac{V}{A}\times \frac{d_{c}}{d_{t}}\times \frac{T_{0}}{T}\times \frac{P}{P_{0}}\times 60\times 1000$$

where F represents the N_2_O emission flux (μg·m^−2^·h^−1^) or the CO_2_/CH_4_ emission flux (mg·m^−2^·h^−1^); ρ_0_ represents the gas density under standard conditions (g·L^−1^); V represents the effective gas volume inside the closed chamber (m^3^); A represents the soil surface area inside the chamber (m^2^); d_c_/d_t_ represents the rate of change over time in the target gas component within the closed chamber, with the rate of change for greenhouse gases expressed in (ppb·min^−1^) and that for CO_2_ and CH_4_ expressed in (ppm·min^−1^); T_0_ and P_0_ represent the standard temperature (273.15 K) and standard atmospheric pressure (1.01 × 10^5^ Pa), respectively; and T and P represent the average temperature (K) and pressure (Pa), respectively, inside the chamber at the time of gas sampling.

### 2.5. Statistical Analysis of the Data

The data were analyzed and processed using Microsoft Excel 2021 (Microsoft Corporation, Redmond, WA, USA), and the differences between treatment groups were examined using Duncan’s new multiple range test at the 0.05 significance level with IBM SPSS 27.0 software (IBM Corporation, Armonk, NY, USA). The figures were plotted using Origin 2025 (OriginLab Corporation, Northampton, MA, USA).

## 3. Results

### 3.1. Effects of Organic and Inorganic Fertilizers Combined in Different Ratios on Rice Yield and Nitrogen Use Efficiency

The application of both organic and inorganic fertilizers significantly increased rice yields and nitrogen use efficiency (Table 1). Compared with the conventional 100% chemical fertilizer NPK treatment (CK), the treatments with 30% organic N and 70% chemical N fertilizers with conventional PK fertilizer (T2) and 50% organic N and 50% chemical N fertilizers with conventional PK fertilizer (T3) increased the rice yields by 3.31% and 24.27%, respectively. The treatments without N fertilizer and with conventional PK fertilizer (T1) and the treatment with 100% organic N fertilizer (T4) significantly decreased the rice yields by 34.14% and 9.05%, respectively. Compared with those in the CK treatment group, the partial factor productivity of applied nitrogen (PFPN) and nitrogen use efficiency (NUE) of rice in the T3 treatment group significantly increased (by 24.26% and 71.05%, respectively). Compared with those in the CK treatment group, the PFPN and NUE in the T2 treatment group increased by 3.31% and 9.69%, respectively, but the differences were not significant. Compared with those in the CK treatment, the PFPN and NUE in the T4 treatment group decreased significantly (by 9.05% and 26.49%, respectively). The T3 treatment was determined to be the optimal treatment for increasing rice yields and improving the nitrogen use efficiency in cool regions.

### 3.2. Distribution and Stability of Soil Aggregates with Different Particle Sizes

**Figure 3:**
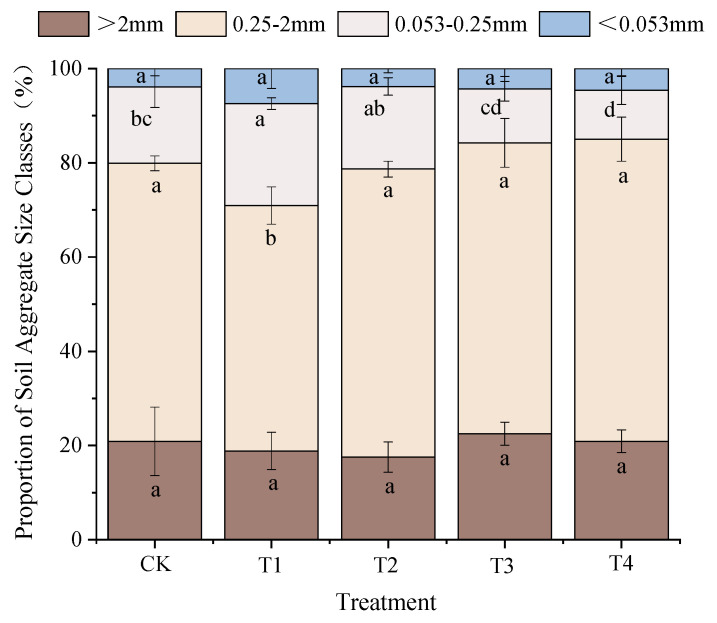
Proportions of water-stable aggregates with different sizes after long-term application of different fertilizer ratios (%). Note: Results with different lowercase letters (means ± standard errors, *n* = 3) in the same column are significantly different according to Duncan’s test (*p* < 0.05).

The proportions of 0.25–2 mm aggregates with different particle sizes in soil treated with varying ratios of organic and inorganic fertilizers were similar (Figure 3 and Table S1), accounting for 52.06–64.08% of the total, whereas aggregates smaller than 0.053 mm accounted for only 3.82–7.45% of the total. Compared with those in the CK treatment group, the proportions of aggregates larger than 2 mm and smaller than 0.053 mm did not significantly differ. After treatment with organic fertilizer (e.g., T2, T3, and T4), the proportions of 0.25–2 mm aggregates in the soil increased by 3.52%, 4.56%, and 8.55%, respectively, compared with those after CK treatment, but the differences were not significant. Compared with that after CK treatment, the proportion of 0.25–2 mm aggregates after T1 treatment significantly decreased by 11.81%. The proportions of 0.053–0.25 mm aggregates in the soil followed the order T1 > T2 > CK > T3 > T4. Compared with the CK treatment, the T1 treatment significantly increased the proportion of aggregates by 33.85%, whereas the T4 treatment significantly decreased this proportion by 36.01%. Compared with those after the CK treatment, the proportions of aggregates after the T2 and T3 treatments increased and decreased, respectively, by 8.60% and 28.90%, but the differences were not significant.

The different fertilization treatments distinctly influenced the soil aggregate stability (Table 2). Compared with that in the CK treatment group, the MWD in each treatment group increased by −9.91%, −3.60%, 4.50%, and 4.50%, respectively. Unlike the T2 treatment group, which was not significantly different from the CK treatment group, the other treatment groups differed from the CK treatment group, but the differences were not significant. The GMD did not significantly differ between the T3 and T4 groups and the CK group. Compared with the CK treatment, the T2 treatment resulted in a difference in the GMD, but this difference was not significant. However, compared with the CK treatment, the T1 treatment significantly decreased the GMD by 5.21%. There were no significant differences in the D_v_ among the treatments.

### 3.3. Effects of Different Organic and Inorganic Fertilizer Ratios on Indicators of Soil Fertility in the Paddy Fields

#### 3.3.1. Effects of Different Organic and Inorganic Fertilizer Ratios on the Physicochemical Properties of Soil in the Paddy Fields

**Figure 4:**
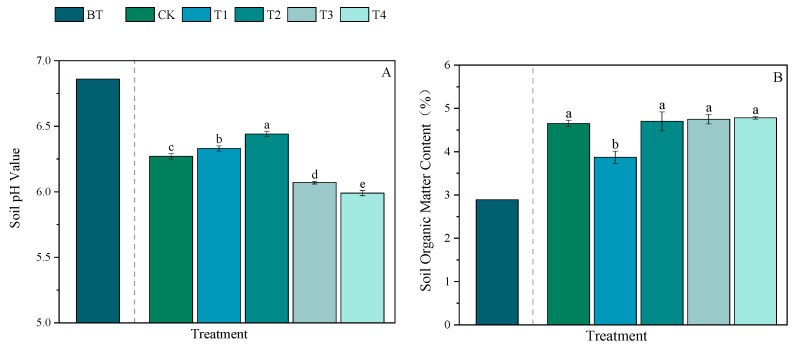

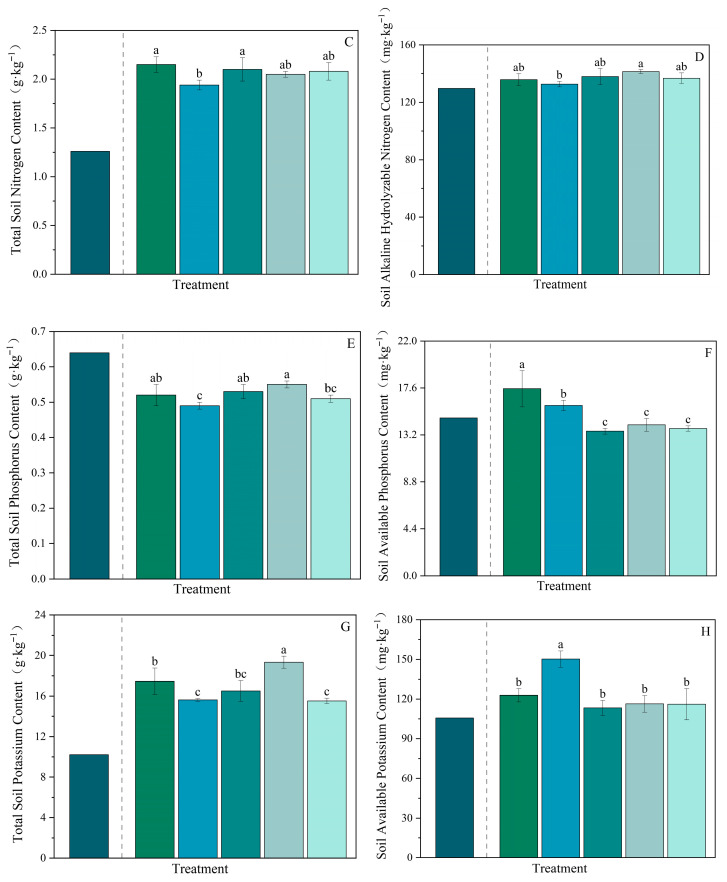
Effects of different fertilizer ratios on the physicochemical properties of soil. (**A**–**H**) represent soil pH, organic matter content, total soil nitrogen content, soil alkaline hydrolyzable nitrogen content, total phosphorus content in soil, soil available phosphorus content, total soil potassium content and soil available potassium content respectively.

Compared with those before the rice trial began (BT), the pH levels of the soils were decreased after all the treatments, with a reduction range of 6.12–12.68%, and significant differences were observed among the treatment groups (Figure 4A and Table S2). Compared with those after the CK treatment, the pH levels after the T1 and T2 treatments significantly increased by 0.96% and 2.71%, respectively, whereas those after the T3 and T4 treatments significantly decreased by 3.19% and 4.47%, respectively. Compared with the values before the experiment, all the treatments increased the soil organic matter (SOM) contents (Figure 4B and Table S2). Among the experimental treatments, except for T1, for which the value was 16.77% lower than that of the CK treatment, there were no significant differences among the remaining treatment groups.

Compared with those before the experiment, the total nitrogen (TN) and alkali-hydrolyzable nitrogen (AN) contents in the soil increased after all the treatments. The increase in soil TN content ranged from 53.97% to 70.63%, and the increase in soil AN content ranged from 2.34% to 9.01% (Figure 4C,D and Table S2). Compared with that in the CK treatment group, the TN content in the T1 treatment group significantly decreased by 9.77%, whereas the TN contents in the other treatment groups did not significantly differ. There were no significant differences in soil AN content between the experimental treatment groups and the CK group.

Compared with those before the experiment, the total phosphorus (TP) contents in the soils in all the groups decreased, with decreases ranging from 14.06 to 23.44% (Figure 4E and Table S2). Unlike the T1 treatment group, for which the level decreased by 5.77% compared with that in the CK treatment group, the remaining treatment groups did not significantly differ from the CK treatment group. The changes in the available phosphorus (AP) contents of the soil compared with those before the experiment ranged from −8.45% to 18.51% (Figure 4F and Table S2). Compared with the CK treatment, all the experimental treatments significantly reduced the AP content by 8.95%, 22.75%, 19.33%, and 21.32%, respectively.

Compared with those before the experiment, the total potassium (TK) and available potassium (AK) contents in the soil were increased by all the treatments. The increase in the soil TK content ranged from 52.16% to 89.61%, and the increase in the soil AK content ranged from 7.24% to 42.14% (Figure 4G,H and Table S2). Compared with that in the CK group, the TK contents in the T1 and T4 treatment groups were significantly decreased by 10.48% and 11.11%, respectively, whereas that in the T3 treatment group was significantly increased by 10.77%. Compared with the CK treatment, the T2 treatment resulted in no significant difference in TK content. Compared with the CK treatment group, the soil AK content in the T1 group was significantly increased by 22.38%, but that in the other treatment groups did not significantly differ.

#### 3.3.2. Effects of Different Organic and Inorganic Fertilizer Ratios on Biological Indicators of Soil in the Paddy Fields

**Figure 5:**
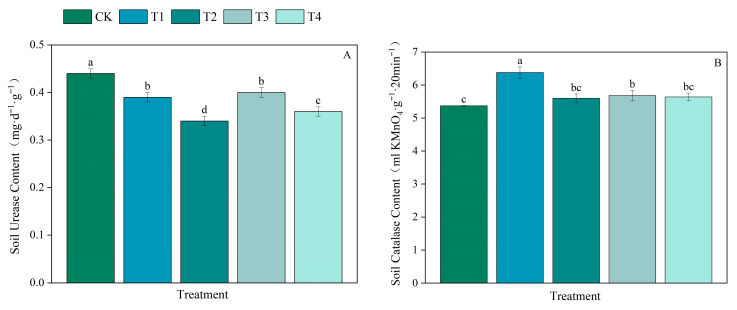
Effects of different fertilizer ratios on the biological indicators of soil. (**A**,**B**) represent soil urease content and soil catalase content respectively. Note: Results with different lowercase letters (means ± standard errors, *n* = 3) in the same column are significantly different according to Duncan’s test (*p* < 0.05).

The different fertilization treatments significantly affected the soil urease (SUE) content (Figure 5A and Table S3). Compared with those in the CK treatment group, the SUE contents in each treatment group decreased significantly, by 11.36%, 22.73%, 9.09%, and 18.18% in the T1, T2, T3, and T4 treatment groups, respectively.

Compared with those after CK treatment, the soil catalase (SCAT) contents increased after all the treatments (Figure 5B and Table S3). Compared with that in the CK treatment group, the SCAT contents in the T1 and T3 treatment groups increased significantly (by 18.81% and 5.77%, respectively). Moreover, those in the T2 and T4 treatment groups increased (by 4.28% and 5.03%, respectively), but the difference was not significant.

### 3.4. Effects of Different Organic and Inorganic Fertilizer Combinations on the Environmental Indicators of Soil in the Paddy Fields

#### 3.4.1. Effects of Different Organic and Inorganic Fertilizer Ratios on Heavy Metal Accumulation in Soil in the Paddy Fields

**Figure 6:**
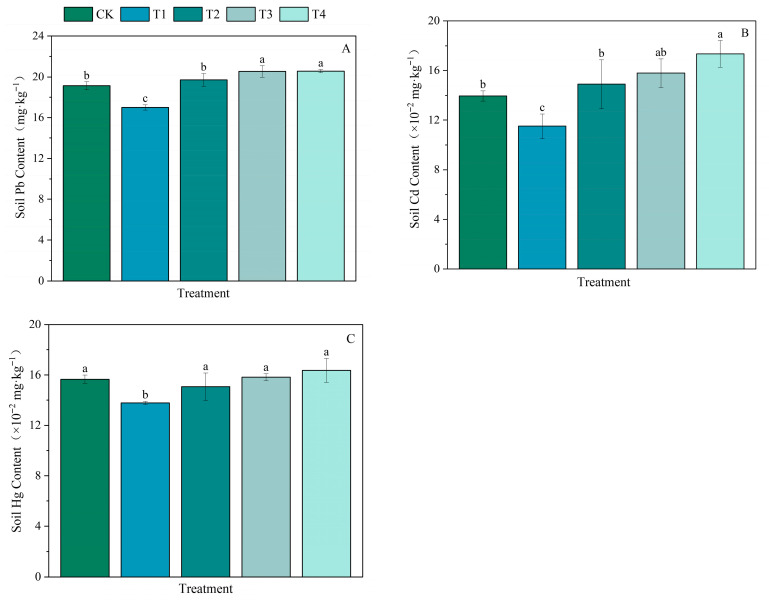
Effects of different organic and inorganic fertilizer ratios on the heavy metal contents in soil in the paddy fields. (**A**–**C**) represent soil Pb content, soil Cd content and soil Hg content respectively. Note: Results with different lowercase letters (means ± standard errors, *n* = 3) in the same column are significantly different according to Duncan’s test (*p* < 0.05).

Different fertilization treatments significantly affected the accumulation of the heavy metals lead (Pb), cadmium (Cd), and mercury (Hg) in the soil of the paddy fields (Figure 4 and Table S4). Compared with the soil Pb content in the CK treatment group, that in the T1 treatment group significantly decreased by 11.09%; that in the T3 and T4 treatment groups significantly increased by 7.43% and 7.53%, respectively; and that in the T2 treatment group increased by 3.03%, but the difference was not significant (Figure 6A). Compared with the soil Cd content in the CK treatment group, that in the T1 treatment group significantly decreased by 17.49%, whereas that in the T4 treatment group significantly increased by 24.37%; the soil Cd contents in the T2 and T3 treatment groups increased by 6.81% and 13.19%, respectively, but the differences were not significant (Figure 6B). Compared with that in the CK treatment group, the soil Hg content in the T1 treatment group decreased significantly (by 12.07%), whereas those in the T2, T3, and T4 treatment groups increased by −3.83%, 1.02%, and 4.47%, respectively; however, the differences were not significant (Figure 6C).

#### 3.4.2. Effects of Different Organic and Inorganic Fertilizer Ratios on Greenhouse Gas Emissions from Soil in the Paddy Fields

**Figure 7:**
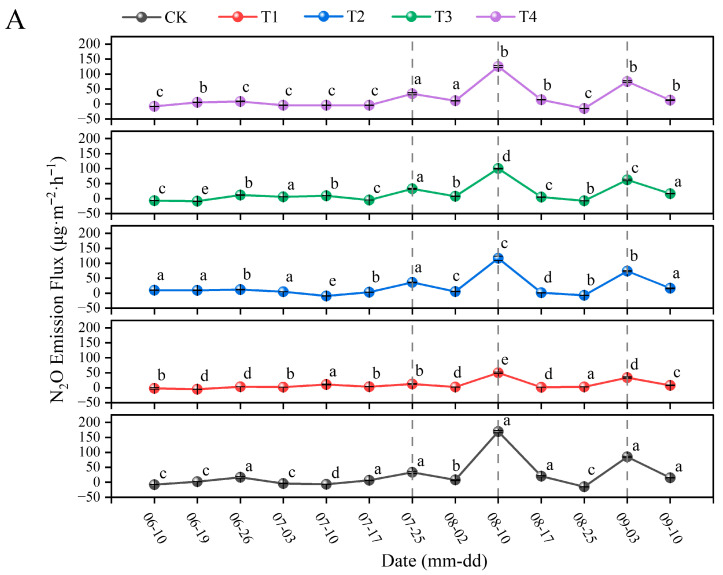

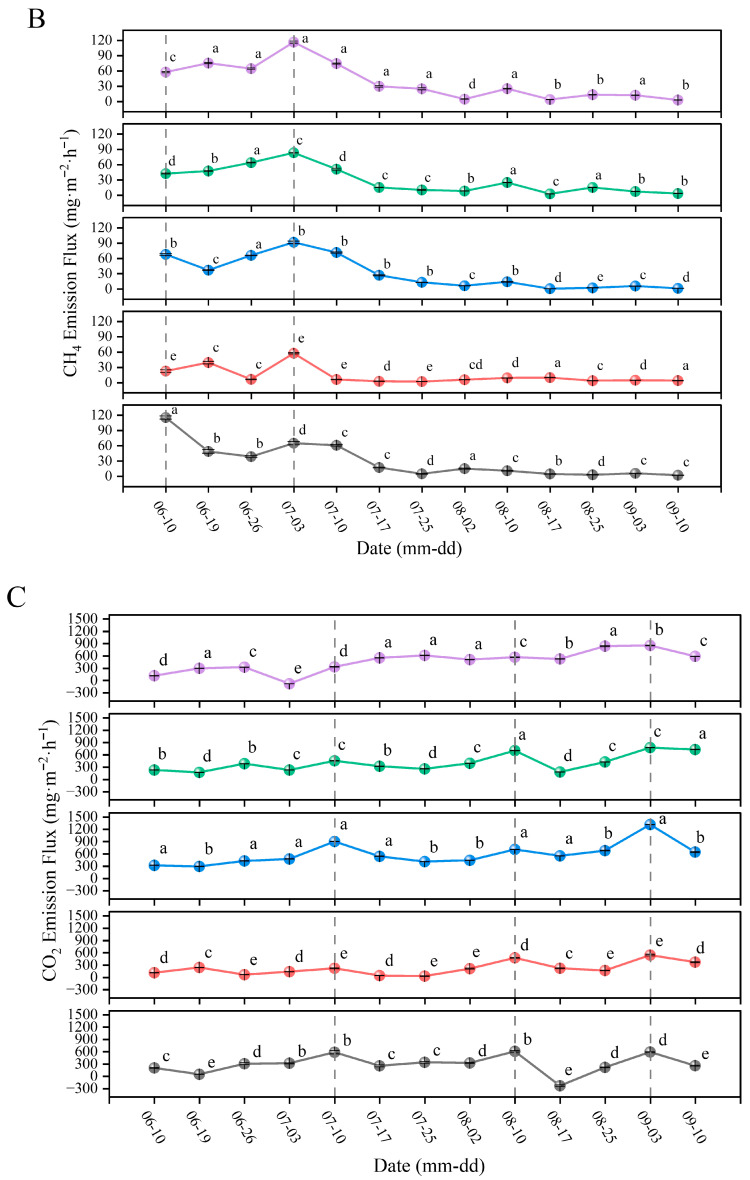
Effects of different fertilizer ratios on soil greenhouse gas emission fluxes. (**A**–**C**) represent the emission fluxes of greenhouse gases N_2_O, NH_4_, and CO_2_ on different dates, respectively. Note: Results with different lowercase letters (means ± standard errors, *n* = 3) in the same column are significantly different according to Duncan’s test (*p* < 0.05).

The greenhouse gas emission fluxes significantly differed among the treatment groups during the rice growth period (Figure 7 and Table S5). The N_2_O emission fluxes among the treatment groups revealed seasonal patterns, with relatively low emissions occurring during the early rice growth period, followed by three distinct emission peaks (Figure 7A and Table S5A). The first N_2_O emission peak occurred on 25 July. Compared with that in the CK treatment group, a significant reduction in N_2_O emission flux (by 61.72%) was observed only in the T1 treatment group, where no nitrogen was applied. The emissions from all the other treatment groups were statistically indistinguishable from those of the CK treatment group. The second N_2_O emission peak occurred on 10 August, which was the period with the greatest N_2_O emissions during rice growth. The N_2_O emissions among the treatment groups decreased in the order of CK > T4 > T2 > T3 > T1. Compared with those in the CK treatment group, the N_2_O fluxes in the T1, T2, T3, and T4 treatment groups decreased by 70.52%, 31.08%, 40.75%, and 26.07%, respectively. The final N_2_O emission peak during the observation period occurred on 3 September, with the greatest N_2_O emissions occurring in the CK treatment group. Compared with those in the CK treatment group, the N_2_O fluxes in the experimental treatment groups decreased by 60.20%, 13.38%, 26.44%, and 11.84%, respectively.

CH_4_ emissions were concentrated mainly during the early rice growth period, with relatively low emissions in the later stages (Figure 7B and Table S5B). The peak periods during the observation period differed among the treatment groups. Following CK treatment, the greatest peak occurred on 10 June, with CH_4_ emissions decreasing in the order of CK > T2 > T4 > T3 > T1; these emissions were 41.21%, 49.77%, 63.39%, and 80.66%, respectively, lower than those of the CK treatment. The peak emissions after the other treatments occurred on 3 July; compared with those after CK treatment, the CH_4_ flux after the T1 treatment was 11.15% lower, while those after the T2, T3, and T4 treatments were 41.75%, 29.01%, and 80.88% greater, respectively.

The different fertilization treatments significantly affected CO_2_ emissions from the paddy fields, but the overall trends were similar, with CO_2_ emission peaks occurring during three concurrent periods (Figure 7C and Table S5C). On 10 July, the CO_2_ emission fluxes after each treatment peaked first during the growth period; compared with that after the CK treatment, the CO_2_ flux after the T2 treatment increased by 54.69%, while those after the other treatments decreased by 62.91%, 21.93%, and 42.81%, respectively. On 10 August, compared with those after the CK treatment, the CO_2_ fluxes after the T2 and T3 treatments increased by 16.65% and 16.17%, respectively, while those after the T1 and T4 treatments decreased by 22.01% and 5.98%, respectively. On 3 September, the CO_2_ emission fluxes after all the experimental treatments peaked, except for the T1 treatment, for which the value decreased by 8.38% compared with that after the CK treatment, and the CO_2_ fluxes after the other treatments increased by 123.84%, 31.93%, and 45.35%, respectively.

In summary, the application of fertilizer containing 50% organic nitrogen can simultaneously reduce N_2_O emissions and mitigate CH_4_ and CO_2_ emissions.

### 3.5. Correlations of Various Production Factors in Paddy Fields Treated with Different Organic Fertilizer-to-Inorganic Fertilizer Ratios

**Figure 8:**
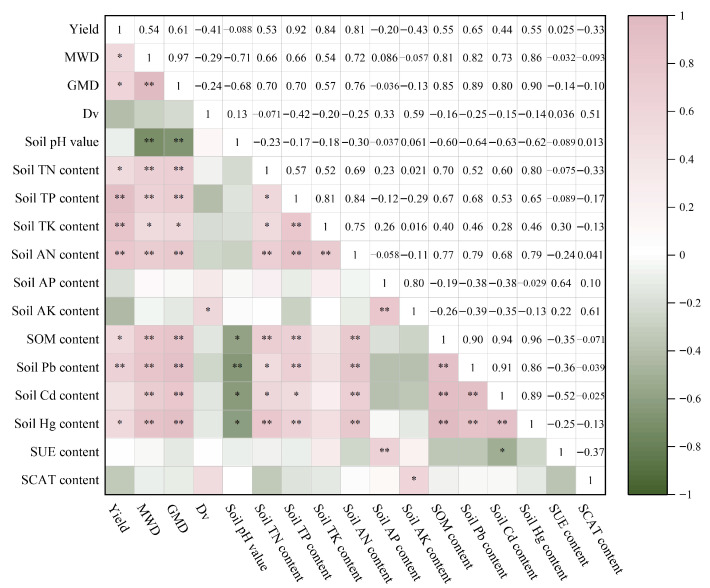
Correlations of various production factors in paddy fields treated with different organic and inorganic fertilizer ratios. Note: * and ** in the figure indicate significant differences (*p* < 0.05) and highly significant differences (*p* < 0.01), respectively. Pink denotes a positive correlation, and green denotes a negative correlation.

Through the use of Spearman’s correlation analysis, we evaluated the correlations of various factors in paddy fields production following treatments with different organic to inorganic fertilizer ratios (Figure 8). The rice yields were strongly positively correlated with the soil TP, TK, and AN contents and the content of the heavy metal Pb; significantly positively correlated with the MWD and GMD values of soil aggregates; and significantly negatively correlated with the soil TN content, SOM content, and Hg content. The MWD and GMD values of soil aggregates were strongly significantly positively correlated with the soil TN, TP, AN, SOM, Pb, Cd, and Hg contents; significantly positively correlated with the TK content; and strongly significantly negatively correlated with the soil pH value. Soil pH was strongly significantly negatively correlated with Pb content and significantly negatively correlated with the Hg, Cd, and SOM contents. The soil AN and SOM contents were strongly significantly positively correlated with the soil Pb, Cd, and Hg contents. The SUE content was strongly significantly positively correlated with the AP content and significantly negatively correlated with the soil Cd content. SCAT activity was significantly positively correlated with the soil AK content.

**Figure 9:**
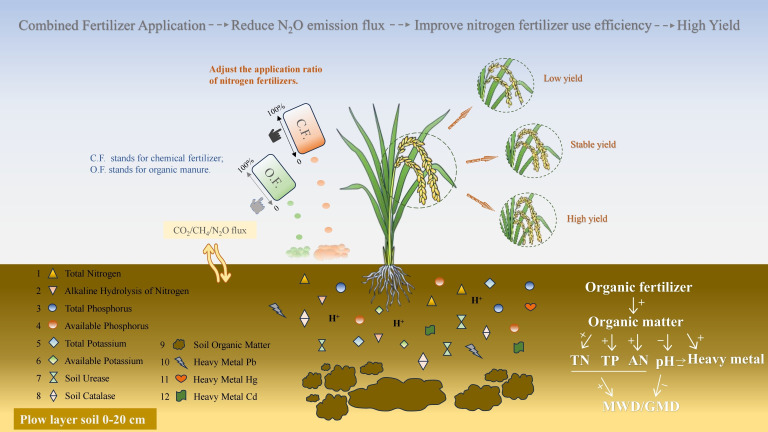
The application of both organic and inorganic fertilizers enhances long-term soil fertility, structural stability, and rice productivity in cool rice-growing regions.

The above findings show that the combination of organic and inorganic fertilizers in the appropriate ratio can increase the nitrogen use efficiency of rice in the cold and cool regions of Northeast China and achieve increased and stable rice yields; moreover, it can effectively improve soil fertility and enhance soil structure stability, which holds profound significance for the environment and the sustainable development of paddy fields (Figure 9).

## 4. Discussion

### 4.1. Effects of Combined Organic and Inorganic Fertilizer Application on Rice Yields and Utilization Efficiency

The results of this study indicate that compared with chemical nitrogen fertilizer alone, combining organic and inorganic nitrogen fertilizers significantly increases the rice yield and nitrogen use efficiency. However, when organic nitrogen entirely replaces chemical nitrogen (100% organic fertilizer), both yield and efficiency decrease. This nonlinear response relationship confirms the existence of a “golden ratio” in organic substitution.

After a chemical nitrogen fertilizer is applied to soil, it rapidly hydrolyzes, providing abundant readily available nitrogen during the two peak periods of nitrogen uptake by rice: the tillering stage and the young spike differentiation stage [39]. This provides the basis for achieving an adequate number of effective panicles and a relatively large panicle size. In contrast, the nitrogen in organic fertilizer exists primarily in organic form and requires microbial mineralization for its release. The nitrogen supply rate is relatively delayed but lasts longer [40,41]. When the organic replacement ratio is 20–50%, chemical nitrogen ensures the “initiating effect” for early tillering, whereas organic nitrogen continuously mineralizes in the middle to late stages to support grain filling, creating a nitrogen supply pattern of “fast at the beginning, steady later” [42,43]. When the organic replacement ratio is 100%, owing to the lack of timely supplementation with readily available nitrogen, the rice experiences “nitrogen deficiency” during early growth, leading to insufficient tillering and lower overall biomass. Although organic nitrogen continues to mineralize later, the critical window for yield formation has already passed, making it difficult to compensate for the loss of yield caused by insufficient early growth. However, this result does not negate the long-term value of organic agriculture; in contrast, it highlights the limitations of 100% organic substitution for seasonal production as well as the necessity of identifying the optimal ratio within an intensive and sustainable production system. With respect to soil fertility enhancement and agricultural sustainability, complete reliance on organic fertilizers, which only mineralize at rates of 20–30%, is unlikely to meet the nitrogen demand required for high crop yields in the short term. A 50% replacement ratio represents the optimal choice that allows this experimental platform to achieve both stable, high yields and improved nitrogen use efficiency.

On the basis of the conclusion that a 50% replacement ratio was optimal in this experiment, future research should integrate ^15^N isotope tracing techniques to clarify the transformation characteristics of fertilizer and soil nitrogen within soil nitrogen pools under conditions with different replacement ratios, as well as the temporal differences in nitrogen uptake by crops, to develop a mechanistic model to guide precise organic substitution strategies [44].

### 4.2. Effects of the Combined Application of Organic and Inorganic Fertilizers on Soil Aggregates and Structural Stability in Paddy Fields

Soil aggregates function as reservoirs of nutrients, and the distribution characteristics of aggregates with different sizes are important indicators for evaluating soil fertility. Microaggregates primarily accumulate in unfertilized soils, whereas fertilization can significantly increase the formation of macroaggregates, with the MWD of soils treated with organic fertilizers being significantly greater than that of untreated soils [45]. Various scholars have indicated that the soil organic matter content is the primary factor that influences the stability of water-stable aggregates [46,47]. Pucetaite et al., via the use of advanced imaging techniques, reported that the input of carbon-rich organic matter increases the proportion of aggregates larger than 0.25 mm, and this phenomenon may be related to fungal growth and hyphal entanglement [48]. The application of organic fertilizers, while increasing labile organic carbon components, increases the number of large organic molecules, such as cellulose, polysaccharides, and humic acids, thereby promoting aggregate formation and increasing aggregate stability [49]. A 10-year field fertilization experiment in Northeast China revealed that the application of organic fertilizers promotes the breakdown of large aggregates (>5 mm) and the aggregation of microaggregates (<0.25 mm), thereby increasing the proportion of medium-sized aggregates (0.25–5 mm) in the soil; however, the differences in aggregate stability after different treatments were not significant [50]. Compared with combined organic–inorganic treatments, the treatment with organic fertilizer alone can more effectively supplement soil nutrients and increase the content and water stability of large soil aggregates [51]. In an experiment that lasted more than 70 years, it was demonstrated that the application of both chemical fertilizers and farmyard manure was more effective than either alone in terms of improving aggregate formation and increasing the organic carbon and nitrogen contents of aggregates [52]. The D_v_s of soil aggregates can be used to effectively describe the stability of aggregates and changes in soil structure that are associated with nutrient succession. The D_v_s of microaggregates are significantly positively correlated with the contents of clay-sized particles (<0.002 mm) and negatively correlated with the contents of medium- and large-sized aggregates [53].

In this experiment, compared with the CK treatment, the nitrogen-free treatment (T1) significantly reduced the proportion of 0.25–2 mm aggregates and aggregate stability, indicating that nitrogen fertilizer plays an important role in maintaining soil structure. Except for soils that received the T1 treatment, the soils that received nitrogen fertilization exhibited increased proportions of soil aggregates larger than 0.25 mm, although there were no significant differences among these treatment groups. Additionally, the MWD and GMD of the soils treated with nitrogen did not significantly differ from those in the soil treated with CK, which is consistent with the findings of the 10-year experiment in Northeast China described above [50]. There is no significant difference in soil D_v_, likely because D_v_ is primarily influenced by the proportions of microaggregates, which do not vary significantly among treatments [54]. The lack of significant differences in soil structure and stability among the nitrogen treatments may be attributed mainly to the long-term cumulative effects of organic fertilizer amendments. Therefore, in the long term, targeted studies are still needed to systematically monitor the dynamic changes in soil aggregate structures, thereby fundamentally improving soil physical health.

### 4.3. Effects of Combined Organic and Inorganic Fertilizer Application on Soil Fertility Indicators in Paddy Fields

Organic fertilizers contain large amounts of various mineral nutrients and active substances. When exogenous organic materials are applied to the soil, they can alleviate the stress caused by long-term chemical fertilizer use, effectively promoting the transformation of soil nutrients, reshaping microbial community structures, and enhancing soil enzyme activities, thereby improving overall soil fertility [55]. The use of organic materials can effectively increase soil carbon and nitrogen accumulation, with the amounts of total nitrogen, alkaline hydrolyzable nitrogen, nitrate nitrogen, and ammonium nitrogen in paddy fields increasing annually [56]. Additionally, the availability of soil nitrogen is significantly positively correlated with the content of organic carbon in soil. In this study, all the fertilization methods significantly increased the soil organic matter content. Correlation analysis revealed that the accumulation of organic matter was extremely significantly positively correlated with the total nitrogen, total phosphorus, and alkaline hydrolyzable nitrogen contents in the soil. In contrast, soil pH was significantly negatively correlated with the organic matter content, with soil pH decreasing as the organic matter content increased.

#### 4.3.1. Effects of Applying Both Organic and Inorganic Fertilizers on pH and Enzyme Activities of Soil in Paddy Fields

This study demonstrated that the application of organic fertilizer with reduced chemical fertilizer significantly lowered the pH of the soil in the paddy fields. As the amount of bioorganic fertilizer increases, the degree of soil acidification intensifies [57]. This phenomenon may occur due to the introduction of many microorganisms via organic fertilizers, because these microorganisms stimulate the decomposition of existing soil organic matter, producing substantial amounts of organic acids and carbon dioxide, and thereby reducing soil pH [58]; this phenomenon may occur due to the increased reductive nature of the soil environment of flooded paddy fields, causing hydrogen peroxide accumulation and reducing the soil pH [59]. Soil pH governs nutrient availability, organic carbon content, and other key soil properties [60]. Changes in soil pH can affect soil nutrient transformation by influencing soil microbial and enzymatic activities. Numerous studies have suggested that the application of organic fertilizers may be more beneficial for maintaining soil pH at an optimal level for nutrient utilization [61,62]. Compared with inorganic fertilizers, organic fertilizers significantly increase enzyme activities and microbial communities. However, compared with samples without fertilization, those with fertilization have been reported to have lower soil pH levels. Lower pH levels are associated with reduced urease contents, possibly because acidic conditions inhibit microbial enzyme secretion and heavy metal release, degrading enzyme activity and microbial activity [63]. In areas where integrated water and fertilizer management practices are used, compared with chemical fertilizer alone, the combination of organic and inorganic liquid fertilizers significantly increased the activities of multiple enzymes and microbial populations in different soil layers. The performance improved with increasing organic fertilizer replacement ratios (70–100%) [64]. Reportedly, an organic fertilizer substitution rate of 20% can maintain crop yields, improve soil nutrient levels and enzyme activities, promote the growth of beneficial soil bacteria, suppress soil pathogens, and optimize the microbial community structure [65]. Findings from long-term experiments indicate that a 30% substitution of organic nitrogen fertilizer for chemical nitrogen fertilizer helps maintain microbial community stability; in contrast, 50% substitution could inhibit the enzymatic activities of key microbial species [66]. In this experiment, compared with the application of a conventional chemical fertilizer, the application of both organic and inorganic fertilizers reduced urease activity and increased catalase activity. Notably, following the 50% organic nitrogen fertilizer treatment, enzyme activity was not negatively impacted by excessively high or low urease activity, and a moderate increase in catalase activity was conducive to improving soil environmental conditions.

#### 4.3.2. Effects of Combined Organic and Inorganic Fertilizer Application on Nutrients in the Soil in the Paddy Fields

Organic fertilizer functions as a nutrient reservoir, continuously releasing nutrients through mineralization. Compared with the application of organic fertilizer or chemical fertilizer alone, the application of both chemical fertilizer and organic fertilizer can increase crop productivity while better balancing the soil nutrient supply. The results of this experiment indicate that all the fertilization treatments increase the contents of soil organic matter, total nitrogen, total potassium, alkaline hydrolyzable nitrogen, and available potassium. However, the extent to which each treatment improved the soil nutrient contents compared with chemical fertilizer alone varied. After harvest, compared with soils that were treated with chemical fertilizer alone, the soils that were treated with organic and chemical fertilizers in different ratios did not significantly differ in terms of organic matter, total nitrogen, or alkaline hydrolyzable nitrogen contents. A possible reason is that chemical fertilizer alone provides sufficient mineral nutrients for crops, significantly increasing crop biomass. As a result, crops return organic matter to the soil through root exudates and postharvest root residues, increasing total organic matter content. The applied nitrogen from chemical fertilizer can be directly absorbed by crops, while some is lost through leaching and volatilization. In soils treated with organic fertilizer, microorganisms consume fixed organic nitrogen while carbon sources are decomposed, and the slowly released nitrogen can be absorbed by crops in large quantities during their vigorous growth period. Therefore, after harvest, the nitrogen levels across the different fertilization treatments were similar.

Nitrogen fertilizer promotes the absorption of potassium by plants. Research has indicated that when nutrient levels are equal, the combined use of organic and inorganic fertilizers not only reduces potassium fixation in the soil but also increases the potassium supply capacity and intensity of the soil [67,68]. In this experiment, the application of both organic and inorganic fertilizers significantly increased the total potassium content; however, the application of nitrogen fertilizer did not significantly affect the soil available potassium content. A possible reason is that acidic soils promote the conversion of available potassium to slowly available potassium pools. Reducing dependence on chemical potassium fertilizers in agricultural production can lay a foundation for sustainable high crop yields and soil health [69].

Organic fertilizers contain relatively large amounts of organic phosphorus, and soil organic matter can reduce the fixation of inorganic phosphorus and promote its decomposition. Numerous studies have shown that the long-term application of organic together with inorganic fertilizers significantly increases soil available phosphorus contents [70,71]. In this experiment, compared with those before the experiment, the total phosphorus contents after the different fertilization treatments decreased, which does not mean that phosphorus disappeared from the soil. In addition to the removal of large amounts of phosphorus by crops for energy supplementation, a possible reason for the decrease in total phosphorus contents is the formation of insoluble iron–aluminum phosphate precipitates under acidic conditions, where phosphorus binds to iron (Fe^3+^) and aluminum (Al^3+^) ions in the soil [72]. With respect to the treatments with chemical nitrogen fertilizer alone and those without chemical nitrogen fertilizer, when conventional phosphorus and potassium fertilizers were applied, the available phosphorus contents after the experiment were increased compared with the pre-experiment levels. In soils treated with chemical nitrogen fertilizer alone, crops show high phosphorus fertilizer utilization efficiency in the early growth stages, but in the middle to late stages, crops experience premature senescence or reduced root activity, resulting in insufficient phosphorus uptake and large amounts of residual available phosphorus remaining in the soil [73]. Conversely, in soils subjected to a long-term absence of nitrogen fertilizer but regular application of phosphorus and potassium fertilizers, the available phosphorus content may increase; this may occur because the fixation of soil phosphorus reaches saturation, while plants absorb less available phosphorus because of nitrogen deficiency [74], leading to continuous accumulation and increased in available phosphorus levels in the soil during the growing period. In soils treated with organic fertilizers, the decreased available phosphorus contents may be attributed to the increased numbers of microorganisms that decompose and utilize phosphorus in paddy fields, where the processes of phosphorus activation and absorption offset each other, causing a temporary decrease in available phosphorus [75]. The available phosphorus content in soil is the result of a dynamic balance between microbial assimilation (uptake into cells) and dissolution activation (substances released to mobilize phosphorus). The effects of different fertilization rates on soil nutrients require longer-term experimental investigation.

After long-term fertilization, both the ratio of organic to inorganic fertilizers and the duration of application significantly influence soil fertility. In the short term, maintaining a proportion of organic fertilizer below 50% is effective for soil enrichment and yield stabilization. As the enrichment period extends, nutrients gradually accumulate, allowing for an appropriate increase in the proportion of organic fertilizer [76]. Research has indicated that a 15% substitution rate with organic nitrogen results in optimal overall improvement in soil nutrient status [77]. Another study revealed that replacing 50% of chemical fertilizer nitrogen with manure nitrogen significantly increased the contents of total nitrogen, alkaline hydrolysable nitrogen, and organic carbon in soil [78]. Furthermore, a multiple linear regression study on black soil demonstrated that as the proportion of organic fertilizer relative to chemical fertilizer increases, soil nutrient levels initially increase but then decrease. Among these, a 30% substitution of nitrogen fertilizer with organic fertilizer was identified as the most suitable ratio for promoting crop yields while improving soil nutrient conditions [79]. A separate 30-year soil enrichment experiment revealed that the optimal ratio of organic and inorganic fertilizers varies with the initial soil fertility. For medium- to low-fertility paddy fields, a 30% organic proportion was significantly effective at improving soil fertility, whereas for high-fertility paddy fields, the optimal proportion ranged from 50% to 70% [80].

On the basis of the synthesis of these findings, the present experiment revealed that a 50% organic nitrogen substitution ratio is the most effective for enhancing soil fertility under the studied conditions.

### 4.4. Effects of the Combined Application of Organic and Inorganic Fertilizers on the Environmental Indicators of Soils in Paddy Fields

To increase crop production and optimize fertilization strategies, one should focus not only on the apparent utilization rate of crops but also on the environmental costs of nutrient loss. Different fertilization treatments result in varying nutrient losses through pathways such as nitrous oxide emissions and leaching, and they may influence the emissions of other greenhouse gases and pose environmental risks, such as soil heavy metal contamination.

#### 4.4.1. Effects of the Combined Application of Organic and Inorganic Fertilizers on Greenhouse Gas Emissions in Paddy Fields

The impact of organic fertilizer substitution on greenhouse gas emissions appears to depend on the ratio. Field observations in black soil farmland areas indicated that a 25% replacement of inorganic fertilizer with organic fertilizer significantly promoted N_2_O emissions. However, when the organic nitrogen replacement ratio reached 50%, N_2_O emissions decreased [81]. This study revealed that N_2_O emissions varied across different periods. However, during the vigorous growth stage, when N_2_O emissions reached significant peaks, the N_2_O flux in paddy fields that were treated with a combination of organic and inorganic fertilizers was significantly lower than that in fields that were treated with chemical fertilizers alone, with the 50% organic fertilizer combined with chemical fertilizer treatment resulting in a particularly notable reduction in emissions. This complexity is echoed in the literature, with a global field survey indicating that nitrogen-rich organic soils under warm, well-drained conditions emit more N_2_O as soil nitrate concentrations and temperatures increase [82]. Furthermore, soils with greater organic matter contents generally possess greater denitrification potential, which can favor N_2_O production [83]. Mechanistically, research has suggested that combining organic and inorganic fertilizers can mitigate N_2_O emissions and nitrogen loss by inhibiting urease activity, suppressing nitrification processes, and reducing the amount of available substrates for denitrification [84].

Organic fertilizers, which serve as external sources of carbon and energy, can directly stimulate soil microbial and root respiration. The gas emission dynamics markedly shift under different soil conditions. Under anaerobic environments, aerobic microbial respiration is suppressed, slowing the decomposition of soil organic matter and thus reducing CO_2_ emissions. Conversely, these conditions significantly promote CH_4_ emissions because of the high activity of methanogenic bacteria, which utilize soil organic carbon to produce methane [85]. The peak CH_4_ flux is closely associated with the addition of crop residues and organic fertilizers to the soil, with the increased input of organic carbon being the most critical factor driving methane emissions [86]. The results of this study indicated that the peak periods of CH_4_ emissions were concentrated mainly during the early growth stages of rice. During these stages, owing to the incomplete decomposition of the previous crop residues, the CH_4_ emission intensity after treatment with chemical fertilizer alone was relatively high. However, as growth progressed and the proportion of organic fertilizer replacing chemical fertilizer increased, the continuous accumulation of exogenous organic carbon inputs in the soil significantly promoted CH_4_ production and emission fluxes.

The differences in CO_2_ emissions were observed to vary across different rice growth stages. In general, the greater the amount of organic matter that is applied, the higher the CO_2_ emissions from the soil [87]. This result occurs because nutrient mineralization and CO_2_ release occur simultaneously. For instance, practices such as drainage can create peaks in nutrient availability, but these often occur at the cost of substantial soil organic carbon loss through CO_2_ emissions [88,89]. Moreover, during the middle and late stages of crop growth, one of the main causes of CO_2_ emissions is the vigorous activity of crop roots. Root exudates can act as “primers” to stimulate microbial mineralization of soil organic matter and residual carbon from organic fertilizers. This rhizosphere priming effect is stronger in the presence of organic materials, leading to additional CO_2_ release [90]. A higher soil respiration rate does not necessarily indicate a degradation of the soil carbon pool. Instead, a higher respiration flux reflects active biological metabolism and rapid nutrient cycling within the system, providing a sustained nutrient supply for grain filling. From the perspective of soil carbon balance, evaluating the dynamic relationship between “carbon input (organic fertilizer applied and root residues)” and “carbon output (CO_2_ emissions)” is key. Previous studies have shown that under conditions with suitable carbon-to-nitrogen ratios, although CO_2_ emissions increase, the efficiency of new soil carbon sequestration increases, establishing a win–win situation of both increased carbon sequestration and increased crop yield [91,92]. Therefore, on the basis of this long-term experimental platform, future research can focus on assessing the coupled mechanisms of the “priming effect–nutrient release–carbon stabilization” under conditions with different organic and inorganic application ratios, providing theoretical support for the synergistic development of agricultural carbon sequestration, emission reduction, and food security.

#### 4.4.2. Effects of the Combined Application of Organic and Inorganic Fertilizers on Heavy Metal Accumulation in Paddy Fields

In terms of heavy metal control, the application of organic fertilizer has a dual effect. The combined application of organic fertilizer not only reduces the inputs of exogenous heavy metals by partially replacing chemical fertilizers but also effectively decreases the bioavailability of existing heavy metals in the soil through the complexation and adsorption of organic matter, thereby inhibiting their migration to crops [93]. However, many studies have reported that long-term application of chemical fertilizers alone or in combination with organic fertilizers may lead to the accumulation of heavy metals in the 0–20 cm plow layer, and excessive fertilizer application ratios can further increase both the total and bioavailable contents of heavy metals in the soil [94,95]. Soil heavy metal accumulation occurs not only because organic fertilizers such as livestock and poultry manure may contain relatively high concentrations of heavy metals themselves and become important exogenous sources of soil heavy metals, but also because heavy metals may be related to soil-related environmental factors [96,97]. Soil pH is a key environmental factor that regulates heavy metal behavior; under acidic conditions, heavy metals are more likely to migrate and undergo transformation. The lower the pH is, the easier it is for fixed heavy metals to be released, leading to their migration into environmental media [98]. The observations from this experiment are consistent with this mechanism [98], with a significant negative correlation between the soil heavy metal content and soil pH; in particular, the correlation with the lead content reached an extremely significant level. Compared with the CK treatment, all the treatments in this experiment that involved the application of organic fertilizers resulted in accumulations of soil heavy metals. This phenomenon may have occurred because after the addition of organic fertilizers, the resulting soil conditions (such as organic matter content, pH, and microbial activity) were more favorable for the immobilization of heavy metals than for their degradation or leaching. After long-term continuous application, this immobilization effect can lead to the sustained accumulation of heavy metals in the soil. Importantly, if the environmental conditions of the soil change in the future, these immobilized heavy metals can be reactivated and released again, potentially causing secondary impacts on the ecosystem. Notably, despite the aforementioned accumulation, under the experimental conditions used in this study, the soil heavy metal contents in all the treatment groups (including the combined treatments) were well below national limits and were completely within the safe range [99]. These findings indicate that with a reasonable application approach, the primary benefits of combined organic and inorganic fertilizer application, such as soil improvement and fertility enhancement, outweigh the environmental risks, which are controllable.

Inevitably, there are certain limitations to this study. First, this experiment was a long-term 8-year positioning experiment, and consistent treatment measures were implemented every year to create a stable soil ecosystem through continuous human intervention; however, this paper focuses on systematic observations during the 8th rice-growing season. After 8 consecutive years of the same treatment, the experimental system (including soil physical and chemical properties, the microbial community structure, and the nutrient cycling process of the soil) has stabilized; the effect of treatment has been fully accumulated, and a relatively balanced “legacy effect” has been formed. Therefore, the observations in the eighth year can well represent the response characteristics of the system after stabilization, and provide a key temporal reference for understanding the cumulative effects of long-term positioning measures on soil and crops. However, rice growth and soil processes are still significantly affected by interannual meteorological fluctuations, and one year of data alone is not sufficient to completely reveal the interannual variability in long-term localization treatment effects. Therefore, subsequent studies should include continuous positioning observations with this platform for many years to evaluate the interannual stability of the response patterns of crops and soil. In addition, integrating the studies of soil microbial function, nutrient cycling processes, and root–soil interactions will help to systematically elucidate the biological pathways through which long-term fertilization regulates ecosystem function [88,89,90], providing a strong theoretical and empirical basis for the development of sustainable, region-specific soil management techniques.

In addition, this study has certain limitations in terms of the statistical methods used. Owing to the objective conditions of the long-term positioning experiments, the number of repeats in each treatment was only three (*n* = 3). This setup is understandable in long-term field experiments, but the limited number of replicates may affect the statistical power and reduce the sensitivity for detecting small differences between treatment groups. To this end, we used the more sensitive Duncan new complex polarization method in multiple comparisons to effectively identify differences between treatment groups; however, this test is less robust than others, and the results need to be interpreted with caution. Nevertheless, the main conclusions of this paper are still reliable because of the large range of observed treatment effects and the consideration of the historical trend of long-term experiments. In the future, as data accumulate over time, we will use more rigorous statistical methods to verify the research results.

## 5. Conclusions

In this study, the effects of different proportions of organic and inorganic fertilizers on soil structure, soil fertility and rice growth in paddy fields in the cold and cool regions of Northeast China were systematically explored through 8-year long-term positioning experiments. Compared with the conventional chemical fertilizer treatment (CK), the application of both organic and inorganic fertilizers significantly improved the rice yield and nitrogen use efficiency; notably, the 50% organic fertilizer N and 50% chemical fertilizer N with conventional PK fertilizer (T3) treatment was the optimal combination for achieving high yield and high nutrient efficiency. The effects of organic and inorganic fertilizer treatment on soil aggregate structure and stability did not significantly differ from those of CK, but the application of both organic and inorganic fertilizers promoted the conversion of soil available nutrients to slow-acting nutrients, laying the foundation for sustained high rice yield and good soil health. The application of organic and inorganic fertilizer combinations could decrease soil urease activity, increase catalase activity, and decrease N_2_O emissions, and the peak flux of CH_4_ and CO_2_ emissions increased with increasing organic matter content; however, overall, the treatment with T3 could significantly promote the health of paddy field ecosystems. Based on the above findings, the application of 50% organic fertilizer nitrogen, 50% chemical fertilizer nitrogen and conventional phosphorus and potassium fertilizer can aid in achieving the comprehensive goals of high and stable rice yields, soil structure optimization, fertility improvement and environmental coordination in the cold and cool regions of Northeast China, and this treatment is suitable for use as the recommended fertilization scheme for paddy fields in this region.

## Figures and Tables

**Table 1:** Effects of different fertilizer ratios on rice yields and nitrogen use efficiency.

Treatment	Yield (kg hm^−2^)	PFPN	NUE
CK	8024.97 ± 266.41 b	53.50 ± 1.78 b	18.27 ± 1.78 b
T1	5284.95 ± 523.88 d	————	————
T2	8290.42 ± 185.01 b	55.27 ± 1.23 b	20.04 ± 1.23 b
T3	9972.53 ± 79.21 a	66.48 ± 0.53 a	31.25 ± 0.53 a
T4	7298.84 ± 175.73 c	48.66 ± 1.17 c	13.43 ± 1.17 c

Note: Results with different lowercase letters (means ± standard errors, *n* = 3) in the same column are significantly different according to Duncan’s test (*p* < 0.05).

**Table 2:** Effects of organic and inorganic fertilizers combined in different ratios on indicators of paddy soil aggregate stability.

Treatment	MWD (mm)	GMD (mm)	D_v_
CK	1.11 ± 0.12 ab	0.96 ± 0.04 a	2.13 ± 0.11 a
T1	1.00 ± 0.06 b	0.91 ± 0.03 b	2.29 ± 0.15 a
T2	1.07 ± 0.04 ab	0.95 ± 0.01 ab	2.13 ± 0.05 a
T3	1.16 ± 0.06 a	0.98 ± 0.02 a	2.11 ± 0.17 a
T4	1.16 ± 0.04 a	0.98 ± 0.02 a	2.14 ± 0.10 a

Note: Results with different lowercase letters (means ± standard errors, *n* = 3) in the same column are significantly different according to Duncan’s test (*p* < 0.05).

## Data Availability

The original contributions presented in this study are included in the article/Supplementary Materials. Further inquiries can be directed to the corresponding authors.

## Supplementary Materials

The following supporting information can be downloaded at https://www.mdpi.com/article/10.3390/plants15070993/s1, including Table S1: Proportions of water-stable aggregates with various particle sizes after the long-term application of different ratios of organic and inorganic fertilizers (%); Table S2: Physicochemical properties of soils from paddy fields treated with different ratios of organic and inorganic fertilizers (e.g., soil pH, organic matter content, total nitrogen content, alkali-hydrolyzable nitrogen content, total phosphorus content, available phosphorus content, total potassium content, and available potassium content); Table S3: Biological indicators of soils treated with different ratios of organic and inorganic fertilizers (soil urease and catalase content); Table S4: Heavy metal contents in soil from paddy fields after treatment with different ratios of organic and inorganic fertilizers; and Table S5: Soil greenhouse gas emission fluxes after different fertilizer treatments (Table S5A N_2_O; Table S5B CH_4_; and Table S5C CO_2_).
