# Supplementary material for: Long-Term Effects of the Combined Application of Organic and Inorganic Fertilizers on Soil Fertility, Structural Stability, and Rice Productivity in Cool Rice-Growing Regions of Northeast China
Source: Plants (Basel). 2026 Mar 24;15(7):993. doi: 10.3390/plants15070993 (PMC13074732; doi:10.3390/plants15070993)
Supplement: Supplementary file 1 [file plants-15-00993-s001.zip › plants-4126480-supplementary.pdf]

Supplementary Materials

Long-Term Effects of the Combined Application of Organic and Inorganic Fertilizers on Soil Fertility, Structural Stability, and Rice Productivity in Cool Rice-Growing Regions of Northeast China

Yuwei Xin [1,2,†], Benqi Yue [3,†], Xin Zhao [1], Shanlong Li [1],Tao Li [1], Jian Ren [1], Yutong Li [1], Yutong Yang [1,4], Wenze Li [1,4], Kokyo Oh [5], Tiehua Cao [1,*], Xuanhe Liang [1,2,*]

1 Institute of Agricultural Resources and Environment, Jilin Academy of Agricultural Sciences (Northeast Agricultural Research Center of China), Changchun 130033, China; xywyykl2021@163.com (Y.X.)

2 Faculty of Agronomy, Jilin Agricultural University, Changchun 130118, China

3 Jilin Province Green Food Office, Changchun 130022, China; 13756926009@139.com (B.Y.)

4 Agriculture College of Yanbian University, Yanbian Korean Autonomous Prefecture, 133002, China

5 Center for Environmental Science in Saitama, Saitama 347-0115, Japan; o.kokyo@pref.saitama.lg.jp;

* Correspondence: liangxuanhe_2004@163.com (X.L.); caotiehua2002@163.com (T.C.)

† These authors contributed equally to this work.

Table S1 Proportions of water-stable aggregates with various particle sizes after the long-term application of different ratios of organic and inorganic fertilizers (%)

Treatment	＞2mm	2-0.25mm	0.053-0.25mm	＜0.053mm
CK	20.87±7.26 a	59.03±1.58 a	16.16±4.32 bc	3.94±1.52 a
T1	18.85±3.99 a	52.06±3.98 b	21.63±1.23 a	7.45±4.25 a
T2	17.53±3.20 a	61.11±1.68 a	17.55±1.86 ab	3.82±0.94 a
T3	22.50±2.44 a	61.72±5.15 a	11.49±2.62 cd	4.29±2.74 a
T4	20.91±2.42 a	64.08±4.66 a	10.34±2.99 d	4.67±1.55 a

Note: The experiment included five treatments: 100% conventional chemical fertilizer NPK (CK); conventional PK fertilizer without N fertilizer (T1); 30% organic N and 70% chemical N with conventional PK fertilizer (T2); 50% organic N and 50% chemical N with conventional PK fertilizer (T3); and 100% organic N (T4), with the total fertilizer amount kept consistent across treatments. Data in the same column with different lowercase letters (mean ± standard error, n=3) indicate statistically significant differences based on Duncan's test ($P<0.05$). The symbols in the following tables and figures are the same as in this table.

Table S2 Physicochemical properties of soils from paddy fields treated with different ratios of organic and inorganic fertilizers

Treatment	pH	SOM（%）	TN（g • kg^{-1}）	TP（g • kg^{-1}）	TK（g • kg^{-1}）	AN（mg • kg^{-1}）	AP（mg • kg^{-1}）	AK（mg • kg^{-1}）
CK	6.27±0.02 c	4.65±0.07 a	2.15±0.08 a	0.52±0.03 ab	17.46±1.31 b	135.80±4.16 ab	17.54±1.70 a	122.77±4.99 b
T1	6.33±0.02 b	3.87±0.14 b	1.94±0.05 b	0.49±0.01 c	15.63±0.13 c	132.68±1.91 b	15.97±0.48 b	150.24±6.16 a
T2	6.44±0.02 a	4.70±0.22 a	2.10±0.12 a	0.53±0.02 ab	16.51±1.02 bc	137.96±5.60 ab	13.55±0.27 c	113.35±5.67 b
T3	6.07±0.01 d	4.75±0.11 a	2.05±0.03 ab	0.55±0.01 a	19.34±0.59 a	141.33±1.44 a	14.15±0.61 c	116.35±6.29 b
T4	5.99±0.02 e	4.78±0.03 a	2.08±0.09 ab	0.51±0.01 bc	15.52±0.28 c	136.76±3.70 ab	13.80±0.25 c	116.12±11.69 b

Note: SOM, Soil Organic Matter; TN, Total Soil Nitrogen; TP, Total Soil Phosphorus; TK, Total Soil Potassium; AN, Soil Alkaline Hydrolyzable Nitrogen; AP, Soil

Available Phosphorus; AK, Soil Available Potassium.

Table S3 Biological indicators of soils treated with different ratios of organic and inorganic fertilizers

Treatment	Soil Urease Content (mg • d^{-1} • g^{-1})	Soil Catalase Content (ml $KMnO_4$ • g^{-1} • $20min^{-1}$)
CK	0.44±0.01 a	5.37±0.01 c
T1	0.39±0.01 b	6.38±0.17 a
T2	0.34±0.01 d	5.60±0.13 bc
T3	0.40±0.01 b	5.68±0.16 b
T4	0.36±0.01 c	5.64±0.12 bc

Table S4 Heavy metal contents in soil from paddy fields after treatment with different ratios of organic and inorganic fertilizers

Treatment	Soil Pb Content (mg • kg^{-1})	Soil Cd Content (×10^{-2} mg • kg^{-1})	Soil Hg Content (×10^{-2} mg • kg^{-1})
CK	19.12±0.39 b	13.95±0.41 b	15.66±0.33 a
T1	17.00±0.26 c	11.51±1.00 c	13.77±0.12 b
T2	19.70±0.63 b	14.90±1.98 b	15.06±1.10 a
T3	20.54±0.56 a	15.79±1.16 ab	15.82±0.27 a
T4	20.56±0.17 a	17.35±1.08 a	16.36±0.95 a

Table S5 Soil greenhouse gas emission fluxes after different fertilizer treatments

Table S5-A N_2O Emission Fluxes at Different Growth Stages of Rice

Treatment	06-10	06-19	06-26	07-03	07-10	07-17	07-25
CK	-7.73±0.06 c	2.03±0.07 c	16.60±2.82 a	-4.05±1.08 c	-6.62±0.03 d	6.56±0.52 a	33.18±3.58 a
T1	-2.08±2.59 b	-4.95±0.35 d	3.14±0.16 d	2.02±0.25 b	10.90±1.38 a	3.50±0.28 b	12.70±1.20 b
T2	9.82±0.46 a	9.50±0.95 a	12.12±0.68 b	4.90±0.28 a	-9.05±0.44 e	3.33±0.26 b	36.12±1.12 a
T3	-6.96±0.10 c	-8.78±0.60 e	11.76±0.87 b	5.80±0.50 a	9.27±0.21 b	-4.82±0.19 c	32.57±1.42 a
T4	-7.96±0.52 c	5.59±0.55 b	8.50±1.02 c	-4.04±0.33 c	-4.27±0.51 c	-4.25±0.47 c	34.15±3.46 a

Treatment	08-02	08-10	08-17	08-25	09-03	09-10
CK	8.01±1.69 b	169.41±3.66 a	20.44±2.58 a	-15.01±0.72 c	85.07±1.90 a	15.14±0.79 a
T1	2.31±0.37 d	49.95±2.18 e	1.61±0.15 d	2.71±0.35 a	33.86±2.24 d	8.02±1.01 c
T2	5.58±0.48 c	116.76±7.03 c	1.54±0.17 d	-7.18±0.44 b	73.69±1.95 b	16.60±1.41 a
T3	7.56±0.23 b	100.37±0.91 d	4.93±0.57 c	-7.61±0.41 b	62.58±2.39 c	16.26±0.95 a
T4	10.83±0.37 a	125.24±3.25 b	14.24±0.23 b	-15.05±0.33 c	75.00±2.86 b	12.76±1.31 b

Table S5-B CH_4 Emission Fluxes at Different Growth Stages of Rice

Treatment	06-10	06-19	06-26	07-03	07-10	07-17	07-25
CK	114.71±3.06 a	48.13±3.62 b	38.06±1.92 b	64.39±3.10 d	60.34±1.57 c	16.58±0.75 c	4.26±0.81 d
T1	22.18±2.59 e	38.89±2.49 c	5.92±0.47 c	57.21±1.25 e	5.61±0.55 e	2.07±0.33 d	1.64±0.12 e
T2	67.44±1.67 b	36.53±0.59 c	65.47±0.66 a	91.27±2.47 b	71.23±1.18 b	26.44±0.87 b	12.62±0.18 b
T3	41.99±0.94 d	47.27±0.46 b	63.71±0.55 a	83.07±0.17 c	50.80±1.83 d	14.91±0.28 c	10.03±0.95 c

T4	57.62±1.22 c	75.36±1.00 a	64.36±1.10 a	116.47±1.88 a	74.24±0.93 a	29.62±1.72 a	24.89±1.97 a

Treatment	08-02	08-10	08-17	08-25	09-03	09-10
CK	14.37±1.01 a	10.20±0.71 c	4.00±0.19 b	2.36±0.31 d	5.13±0.08 c	1.46±0.10 c
T1	5.22±0.30 cd	8.81±0.14 d	9.36±0.35 a	3.33±0.17 c	4.15±0.18 d	3.46±0.25 a
T2	5.83±0.08 c	13.58±0.74 b	0.21±0.02 d	1.84±0.05 e	5.36±0.20 c	0.84±0.04 d
T3	7.97±0.14 b	24.70±0.13 a	2.19±0.12 c	14.92±0.11 a	7.01±0.11 b	3.15±0.07 b
T4	4.56±0.30 d	24.98±0.68 a	3.64±0.19 b	13.23±0.24 b	12.15±0.37 a	2.88±0.20 b

Table S5-C CO_2 Emission Fluxes at Different Growth Stages of Rice

Treatment	06-10	06-19	06-26	07-03	07-10	07-17	07-25
CK	196.19±13.53 c	40.32±2.25 e	299.94±23.22 d	309.96±10.65 b	580.15±43.93 b	246.96±23.86 c	332.80±22.59 c
T1	107.38±3.16 d	235.89±1.86 c	59.15±3.53 e	134.65±2.46 d	215.18±6.98 e	37.00±0.67 d	24.28±2.99 e
T2	315.81±1.69 a	289.79±2.48 b	423.37±2.10 a	471.95±1.86 a	897.42±2.55 a	534.40±1.43 a	405.68±1.71 b
T3	226.31±1.23 b	168.28±1.46 d	380.90±1.65 b	226.26±1.97 c	452.92±0.45 c	317.25±4.50 b	254.16±1.90 d
T4	113.88±3.08 d	295.12±2.50 a	322.59±2.29 c	-80.16±1.04 e	331.79±4.57 d	550.93±2.81 a	608.98±5.35 a

Treatment	08-02	08-10	08-17	08-25	09-03	09-10
CK	317.66±10.76 d	601.91±19.35 b	-237.96±20.36 e	209.86±6.72 d	585.35±6.66 d	252.05±9.95 e
T1	206.47±12.38 e	469.44±9.89 d	216.16±5.75 c	162.08±2.93 e	536.29±19.29 e	363.89±7.69 d
T2	437.99±0.23 b	702.13±1.67 a	546.19±0.80 a	674.97±4.79 b	1310.27±1.61 a	637.54±11.15 b
T3	389.32±2.26 c	699.22±3.72 a	177.66±1.35 d	422.91±1.13 c	772.27±1.33 c	725.36±2.75 a
T4	508.33±3.64 a	565.89±6.97 c	524.05±3.13 b	836.12±11.03 a	850.81±3.14 b	587.26±1.84 c

Note: The unit of N_2O emission flux is $\mu g \cdot m^{-2} \cdot h^{-1}$, and The unit of CO_2 or CH_4 emission flux emission flux is $mg \cdot m^{-2} \cdot h^{-1}$.
